# Supplementary figures and images for: Clinical and perinatal outcomes of fresh single-blastocyst-transfer cycles under an early follicular phase prolonged protocol according to day of trigger estradiol levels
Source: PeerJ. 2021 Jul 26;9:e11785. doi: 10.7717/peerj.11785 (PMC8320517; doi:10.7717/peerj.11785)

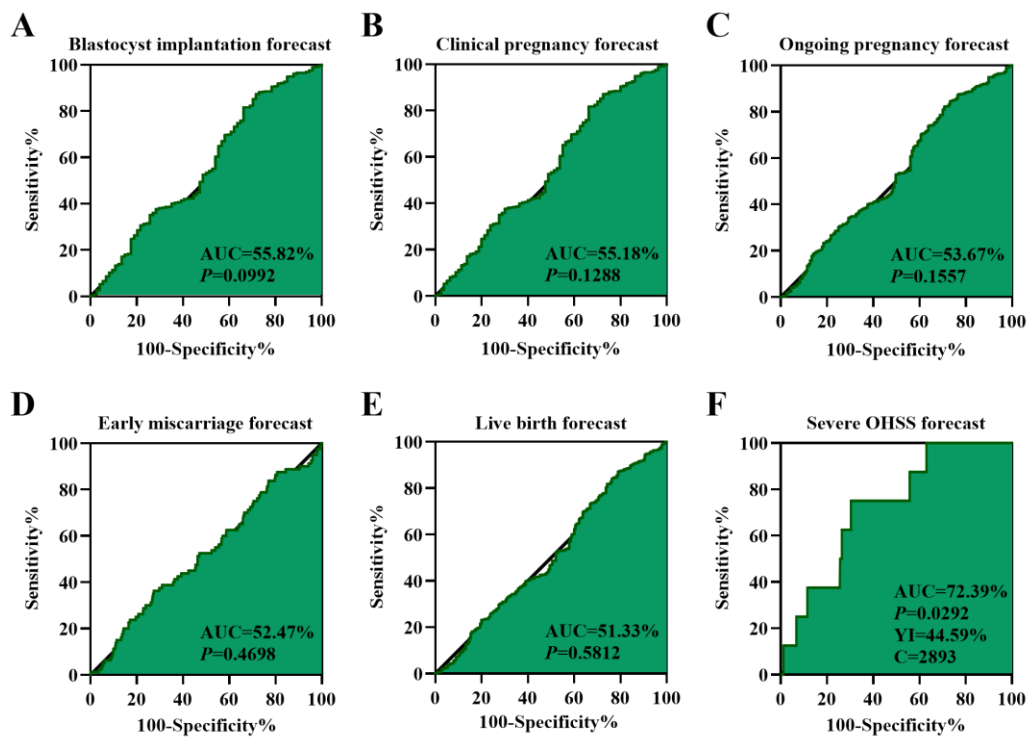

Supplement: Supplemental Information 2 — ROC was used to further analyze the correlation between E2 level on the trigger day and forecasts of fresh single SBT, clinical pregnancy, sustained pregnancy, early abortion, live birth and early-onset severe OHSS. We found that the level of E2 on the trigger day was unpredictable of rates of fresh single SBT, clinical pregnancy sustained pregnancy, early abortion and live birth, that the AUC were from 51.33% to 55.82% with P value greater than 0.05 (Shown in A-E). What is noteworthy is that ROC curve of E2 level and early set-on OHSS had moderate intensity correlation, of which AUC was 72.39%, P value was 0.0292, youden index (YI) was 44.59% and the cut off value of E2 was 2,893 pg/ml with the 75% sensitivity and 70% specificity (Shown in F). [file peerj-09-11785-s002.pdf]
