# Supplementary material for: Clinical and perinatal outcomes of fresh single-blastocyst-transfer cycles under an early follicular phase prolonged protocol according to day of trigger estradiol levels
Source: PeerJ. 2021 Jul 26;9:e11785. doi: 10.7717/peerj.11785 (PMC8320517; doi:10.7717/peerj.11785)
Supplement: Supplemental Information 3 [file peerj-09-11785-s003.docx]

| **Suplementary Table S1 Definitions of different rates** | |  |  |  |  |
| --- | --- | --- | --- | --- | --- |
| **Rates** | **Definition** |  |  |  |  |
| Clinical pregnancy rate | Number of cycles of clinical pregnancy (gestational sac can be seen under B-ultrasound on the 26th day after blastocyst transplantation) / number of transplantation cycles |  |  |  |  |
| Ongoing pregnancy rate | Number of ongoing pregnancy cycles (more than 12 gestational weeks) / number of transplantation cycles |  |  |  |  |
| Embryo implantation rate | Number of gestational sac / number of transferred embryo |  |  |  |  |
| Twin pregnancy rate | Number of twin pregnancy cycles / number of clinical pregnancy cycles |  |  |  |  |
| Monozygotic twin pregnancy rate | Number of one gestational sac cycles (two fetuses share the same gestational sac under ultrasound) / number of clinical pregnancy cycles |  |  |  |  |
| Dichorionic diamnionic rate | Number of two gestational sac cycles (two fetuses have respective gestational sac under ultrasound) / number of clinical pregnancy cycles |  |  |  |  |
| Early abortion rate | Number of early abortion cycles (those whose pregnancy is less than 12 gestational weeks and terminated) / number of clinical pregnancy cycles |  |  |  |  |
| Ectopic pregnancy rate | Number of ectopic pregnancy cycles (ultrasound can see gestational sac outside the uterus) / number of clinical pregnancy cycles |  |  |  |  |
| Live birth rate | Number of live birth cycles / number of transplantation cycles |  |  |  |  |
| Preterm delivery rate | Number of preterm delivery cycles (delivery between 28 and 37 weeks of gestational weeks) / number of clinical pregnancy cycles |  |  |  |  |
| Post-term delivery rate | Number of post-term delivery cycles (the number of delivery cycles that the pregnancy reaches or exceeds 42 weeks) / number of clinical pregnancy cycles |  |  |  |  |
| Cesarean section rate | Number of cesarean section cycles / number of clinical pregnancy cycles |  |  |  |  |
| Incidence rate of birth defect fetus | Number of defect fetuses / total number of fetuses |  |  |  |  |
| Incidence rate of low birth weight infant | Number of low birth weight infants (fetal weight < 2500 g at birth) / total number of fetuses |  |  |  |  |
| Very low birth weight infant rate | Number of very low birth weight infants (fetal weight < 1500 g at birth) / total number of fetuses |  |  |  |  |
| Incidence rate of macrosomia | Number of macrosomia (fetal weight at birth > 4000 g) / total number of fetuses |  |  |  |  |
| Sex ratio at birth | Number of male fetuses versus female girls |  |  |  |  |
| Good quality blastocyst formation rate | Number of good quality blastocysts / number of culturing D3 blastomeres |  |  |  |  |
| Blastocyst formation rate | Number of blastocysts / number of culturing D3 blastomeres |  |  |  |  |
| NICU fetal rate | Number of NICU fetuses / total number of born fetuses |  |  |  |  |
| GDM rate | Number of GDM mothers / number of mothers whho giving birth fetus |  |  |  |  |
| Good quality D3 embyro rate | Number of good quality D3 embyros / number of D3 embryos |  |  |  |  |
| Biochemical pregnancy rate | Number of β-hCG that was less than 10 IU/L / number of transplantation cycles |  |  |  |  |
| Cesarean section rate | Number of cesarean section cycles / clinical pregnancy cycles |  |  |  |  |
| Gestational Hypertension  rate | Number of gestational hypertension mothers / number of mothers whho giving birth fetus |  |  |  |  |
| Placenta previa rate | Number of placenta previa mothers / number of mothers whho giving birth fetus |  |  |  |  |
| PROM rate | Number of PROM mothers / number of mothers whho giving birth fetus |  |  |  |  |
| PPROM rate | Number of PPROM mothers / number of mothers whho giving birth fetus |  |  |  |  |
| NICU=Neonatal intensive care unit, GDM=Gestational diabetes mellitus, PROM=Premature rupture of membranes, PPROM=Preterm premature rupture of membranes | |  |  |  |  |
